# Bilateral Orbital Mass Lesions: a Presentation of Wegener’s Granulomatosis

**Published:** 2011-07

**Authors:** Maryam Aletaha, Mehdi Tavakoli, Mozhgan Rezaei Kanavi, Ali Hashemlou, Shiva Roghaei

**Affiliations:** Ophthalmic Research Center, Shahid Beheshti University of Medical Sciences, Tehran, Iran

A 33-year-old male patient presented with gradual and progressive protrusion of his left eye. He had a history of rhinoplasty for correction of nasal septal deviation 10 years ago. The patient developed intermittent bloody nasal discharge one year after rhinoplasty and underwent diagnostic paranasal sinus endoscopy and maxillary sinus antrostomy and biopsy. Histopathological studies had revealed a granulomatous leukocytoclastic vasculitis compatible with Wegener’s granulomatosis (WG). Treatment with prednisolone 25 mg/day and Endoxan (cyclophosphamide) 100 mg/day was initiated until orbital symptoms and signs appeared, 6 years later. At the time of presentation, uncorrected visual acuity was 20/20 in both eyes without relative afferent pupillary defect. Exophthalmometric readings were 22 and 27 mm on the right and left side, respectively.

In addition, saddle nose deformity was noted (Fig. 1). Mild limitation of ocular movements was present in his left eye. Slit lamp biomicroscopy, tonometry and funduscopy were unremarkable in both eyes. Orbital computed tomography (CT) and magnetic resonance imaging (MRI) scans (Figures 2 and 3) revealed destruction of the nasal septum and bilateral orbital mass lesions.

All laboratory tests including antineutrophil cytoplasmic antibody (cANCA, pANCA), rheumatoid factor, lupus erythematosus cell, C-reactive protein and HLA typing (HLA-B12 and HLA-B5) were negative.

Excisional biopsy of the left orbital mass through a Lynch incision was performed and the mass was completely removed (Fig. 4). Histopathological examination of the specimen confirmed granulomatous vasculitis compatible with WG (Fig. 5). Anti-inflammatory medications were continued. The rheumatologist recommended excisional biopsy of the right orbital mass as well, but the patient declined further intervention.

## DISCUSSION

WG is a systemic disorder that mainly involves the upper and lower respiratory tracts and kidneys. It is characterized by granuloma formation, necrosis and small vessel inflammation on histopathological examination. Some forms of the disease are limited to the respiratory tract without renal involvement. WG is presumed to be autoimmune but its exact nature is unknown; it is believed that infectious agents may trigger the disease. Sinus involvement leads to mucosal thickening, ulceration and recurrent bleeding. Chronic and recurrent inflammation may cause scarification and destruction of the sinuses leading to impaired drainage and normal function. Surgical intervention may be required for treatment of recurrent infections.^1^,^2^

Ocular manifestations such as orbital inflammatory disease, scleritis, episcleritis, and peripheral ulcerative keratitis are major causes of morbidity which occur in more than 50% of patients with WG. Orbital involvement usually presents with proptosis, occasional pain, eyelid edema and limitation of ocular movements and is the most prevalent ocular involvement by WG.^3^ In a large 16-year survey, 140 patients with biopsy-proven WG were examined and orbital disease was reported as the most common ocular involvement in 15% of cases.^4^ Similar results have been reported by other authors.^1^,^5^

Orbital involvement usually present several years after the onset of the disease (as in our patient), however the ophthalmologist may be the first physician who faces a patient with WG. Therefore this condition should always be considered in patients with orbital mass lesions and chronic sinus disease.^1^,^6^ Orbital inflammatory disease in WG may develop as a result of primary granulomatous vasculitis or more commonly following extension of sinus involvement into the orbital cavity. In the latter condition, orbital involvement is usually extraconal but may later extend into the muscle cone.^7^

Despite the high prevalence of orbital granulomatous disease in WG, bilateral involvement is uncommon. Bilateral orbital lesions due to concomitant sinus disease were reported in 2 out of 11 patients by Bullen et al^4^. Differential diagnosis for bilateral orbital mass lesions includes systemic metastasis, lymphoma, leukemia and other types of orbital vasculitis.^8^

Definitive diagnosis of WG is based on tissue biopsy. Histopathological findings include the classic triad of vasculitis, granulomatous inflammation (with or without giant cells) and tissue necrosis; however, the full picture may not be seen in one specimen.^9^

In summary, WG should be considered in patients with uni- or bilateral localized orbital mass lesions especially those with underlying chronic paranasal sinus disease, with or without systemic involvement. Prompt treatment with potent immunosuppressive agents including cyclophosphamide is necessary to improve visual and systemic prognosis.

## Figures and Tables

**Figure 1:**
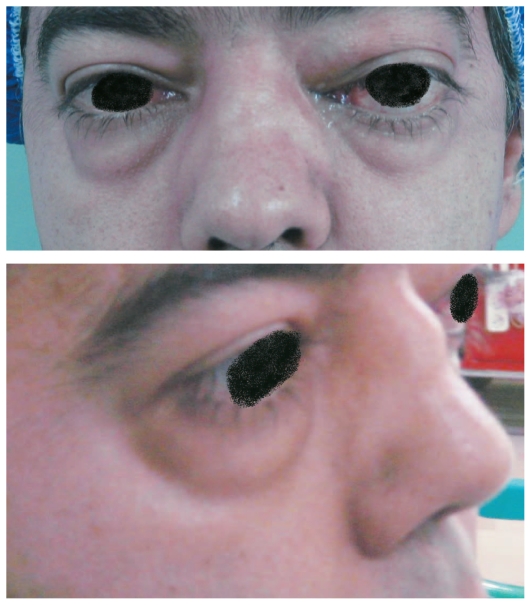
Anterior and lateral appearance of the patient. Note the bilateral proptosis, more marked on the left side, eyelid edema and saddle nose deformity.

**Figure 2:**
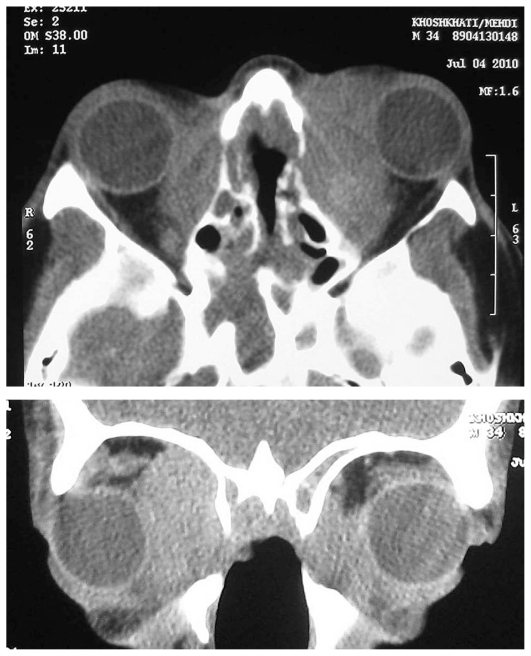
Axial (top) and coronal (bottom) sections on orbital computed tomography. Homogenous extraconal orbital mass lesions are seen on both sides adjacent to the medial walls. Bone erosion and destruction of the medial wall and nasal septum are also noted. The globes are displaced anteriorly and laterally especially on the left side.

**Figure 3:**
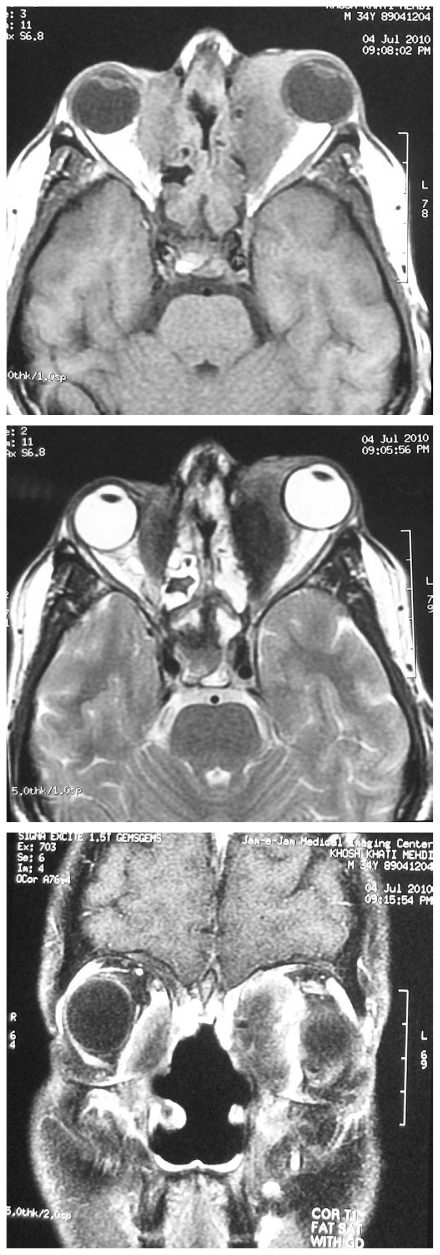
Orbital magnetic resonance imaging shows heterogenous bilateral orbital mass lesions adjacent to the medial walls. The lesions have moderate intensity, similar to muscle on axial T_1_-weighted (top) and low intensity on axial T_2_-weighted images (middle). On coronal fat suppression T_1_-weighted view with contrast (bottom) the lesions show marked heterogenous enhancement. Diffuse mucosal thickening in the paranasal sinuses and absence of the nasal septum are seen in all images.

**Figure 4:**
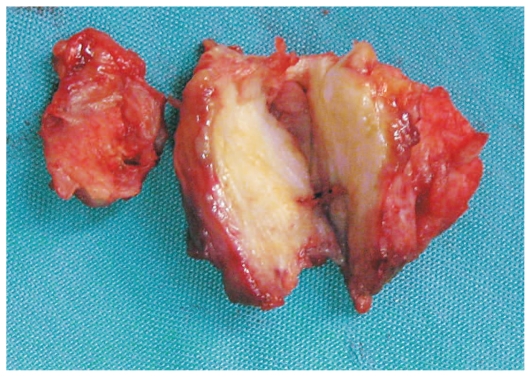
Gross anatomy of the excised mass. Firm consistency and yellowish color of the lesion are similar to that of cartilage.

**Figure 5:**
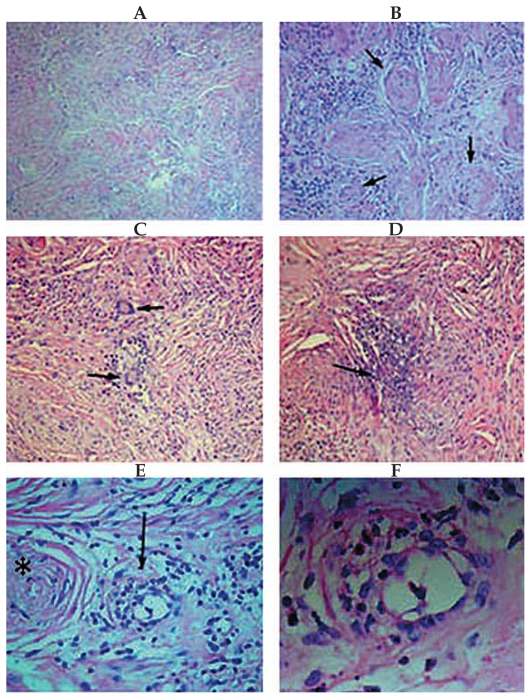
Histopathological examination of the specimen shows evidence of granulomatous vasculitis: (A) Fibrotic and collagenized vascular tufts in which the lumens are occluded with infiltration of acute and chronic inflammatory cells within the collagenous stroma (Hematoxylin & Eosin, ×100). (B) Higher magnification shows occluded vascular lumens (arrows) surrounded by infiltration of inflammatory cells (Hematoxylin & Eosin, ×200). (C) Granulomatous inflammation accompanied by infiltration of Langhans cells, a variety of multinucleated giant cells (Hematoxylin & Eosin, ×200). (D) Small area of necrosis surrounded by acute inflammatory cells in the background stroma (Hematoxylin & Eosin, ×200). (E) Polymorphonuclear cells and eosinophils have infiltrated the vascular wall, compatible with vasculitis (arrow). The lumen of an occluded vessel is visible adjacent to the vasculitis on the left side of the image (asterisk) (Hematoxylin & Eosin, ×400). (F) Higher magnification of the vasculitis shown in figure E (Hematoxylin & Eosin, ×1000).
